# Muskrats as a bellwether of a drying delta

**DOI:** 10.1038/s42003-021-02288-7

**Published:** 2021-06-24

**Authors:** Ellen M. Ward, Katherine A. Solari, Amruta Varudkar, Steven M. Gorelick, Elizabeth A. Hadly

**Affiliations:** 1 Department of Earth System Science, Stanford University, Stanford, CA USA; 2 Department of Biology, Stanford University, Stanford, CA USA

**Keywords:** Riparian ecology, Population genetics, Boreal ecology, Conservation biology, Ecological modelling

## Abstract

Wetlands worldwide are under threat from anthropogenic impacts. In large protected North American areas such as Yellowstone and Wood Buffalo National Parks, aquatic habitats are disappearing and wetland-dependent fauna are in decline^1–3^. Here we investigate population dynamics of an indicator species in Canada’s Peace-Athabasca Delta (“the delta”), a World Heritage Site. Based on population surveys, habitat mapping and genetic data from 288 muskrats, we use agent-based modeling and genetic analyses to explain population expansion and decline of the semi-aquatic muskrat (*Ondatra zibethicus*). Simulations quantify a large population (~500,000 individuals) following flood-induced habitat gains, with decreased size (~10,000 individuals) during drying. Genetic analyses show extremely low long-term effective population size (N_e_: 60–127), supporting a legacy of population bottlenecks. Our simulations indicate that the muskrat population in the delta is a metapopulation with individuals migrating preferentially along riparian pathways. Related individuals found over 40 km apart imply dispersal distances far greater than their typical home range (130 m). Rapid metapopulation recovery is achieved via riparian corridor migration and passive flood-transport of individuals. Source-sink dynamics show wetland loss impacts on the muskrat metapopulation’s spatial extent. Dramatic landscape change is underway, devastating local fauna, including this generalist species even in a protected ecosystem.

## Abstract (web summary)

Ward et al. investigate population dynamics of a wetland indicator species, the semi-aquatic muskrat, within the large and protected area of the Peace-Athabasca Delta in Canada. The authors found indications of rapid population increases associated with flood events, genetic signatures of population bottlenecks, long range dispersal dynamics, and declines in the spatial extent of the muskrat population indicative of the impacts of wetland loss even within a protected ecosystem.

## Introduction

Globally, between 50 and 87% of wetlands are estimated to have been lost since 1700^4^. In the 5500-km^2^ Peace-Athabasca Delta (“the delta”) part of Canada’s Wood Buffalo National Park and a World Heritage Site with “In Danger” status pending, one of the world’s largest freshwater deltas has lost critical habitat. Previous work has attributed drying in the delta to the effects of climate change, upstream hydropower development on the Peace River, or a combination of both drivers^5–8^. This habitat loss is concurrent with a dramatic decline in the delta’s muskrat (*Ondatra zibethicus*) population over the past half century, a trend long reported by local Indigenous trappers^9,10^.

Muskrats serve as an ecohydrologic indicator species. The muskrat shares habitat requirements of other wetland species, including fish and waterfowl, requiring vegetation maintained at early successional stages by flooding. Their numbers rise and fall rapidly with changes in the floodplain as they thrive on near-shore vegetation before declining rapidly in “die-offs”, often attributed to their intensive herbivory^11^. Muskrats are key grazers in this system, influencing succession, diversity and productivity of aquatic and emergent vegetation and are important prey for numerous predators^12^.

Despite their ecological significance, the population dynamics of muskrats at the scale of the floodplain remain poorly characterized. Indigenous land users in the delta have observed ecological changes in the delta that threaten their way of life, including fewer spring flooding events and increased desiccation of wetlands, with a decline in wetland species including the muskrat^9^. For generations, muskrat have served as a source of food, fur and income for land users in the delta^9^. Indigenous knowledge holders and experienced trappers are extremely interested in how ecosystem-scale flooding results in rapid muskrat population increases^9^. Based on satellite remote-sensing temporal mapping of habitat, muskrat surveys, and genetic data from across the delta, we conduct independent investigations using agent-based (individual-based) modeling of muskrats and genetic analyses to reveal a likely mechanism for muskrat eruption and die-off. In addition, we investigate links between eruptive periods in population growth, longer-term drying, and intermittent flooding.

We developed a large-scale (computationally intensive), agent-based model of the muskrat population in the delta that simulates the combined life sequences of the total population ranging from 10,000 to >500,000 individuals for the period 1971–2017, with population survey data available for comparison in 21 years over the period 1970–2017. Life events of individual female muskrat are represented, including dispersal, home range establishment, reproduction, and mortality events, repeating annually. Muskrat reorganize their home range locations in the spring “shuffle” when they search for a mate and new territory for the coming year^12,13^. The total population is twice the number of modeled females^12^. Individual dispersal behavior was represented as a constrained random walk that penalized travel over land and favored travel through hydrologic features such as rivers and lakes.

Temporally dynamic habitat maps were created from an atlas of open water and shoreline habitat built from 30m- to 60m-resolution Landsat satellite imagery available for the period 1972–2017. Canny Edge Detection was used to create maps of land, shoreline and water classes. These maps were used to generate annual habitat suitability maps, consisting of a mosaic of viable riparian habitat regions interspersed with regions of no habitat value to muskrat^11^. Habitat maps were discretized into a modeling grid containing over three million hexagons with individual hexagons of width 60 m (0.31 hectares).

Thirty model realizations were run over the period 1972–2017 to simulate recent periods of eruptive population growth and die-off. Modeling results were compared to population survey records comprised of 21 years of surveys counting muskrat houses at sites across the delta^10^. Dispersal flux, represented as the number of individuals that have migrated through a given location, was mapped as the difference between dispersal flux in 2016 and 2015 for comparison to genetic data. Population sources and sinks, measured in productivity (the number of births minus deaths in a location) were mapped for three successive periods of eruptive population growth (1971–1972, 1996–1997, 2014–2015) and subsequent die-off (1975–1976, 1998–1999, 2016–2017). Model results were then compared to muskrat genetic data.

Microsatellite genetic analyses served as an independent measure of muskrat population history, structure, and dispersal patterns in the delta. Muskrat tissue samples were donated by trappers in the winter of 2015/2016 (hereafter referred to as 2015 samples) and the winter of 2016/2017 (hereafter referred to as 2016 samples), corresponding to habitat conditions mapped in the 2015 and 2016 open water seasons and model output for 2015 and 2016. Nine autosomal microsatellite loci were PCR-amplified (primers from Laurence et al., 2009) in 200 and 88 samples from the 2015 and 2016 trapping seasons, respectively^14^.

Effective population size (N_e_), the number of individuals in an idealized population producing the level of genetic diversity measured from the sample population was estimated using NeEstimator^15^. N_e_ is sensitive to past fluctuations in population size and reflects the harmonic mean of the population size over time.

First-order relatives (parent-offspring or full sibling pairs) were detected by cross referencing output from three different programs: Colony v 2.0.6.5, ML-Relate, and Coancestry^16–18^.

Population structure was assessed using the Bayesian clustering approach implemented in the program STRUCTURE v2.3.4^19^. STRUCTURE identifies subpopulations and probabilistically assigns each sampled individual to one or more subpopulations. Pairwise genetic difference between sites within and between years was assessed by calculating R_ST_ in Arlequin^20^.

An 872 bp region of cytochrome b was also PCR-amplified and sequenced from tissue samples. Number of haplotypes, number of polymorphic sites, and nucleotide and haplotype diversity were calculated using DnaSP v6.11.01^21^. Median joining networks for cytochrome b haplotypes from each year separately as well as both years together were constructed using the program PopArt^22^.

Combined agent modeling and genetic analysis results indicate that the muskrat population in the delta is a metapopulation with individuals migrating preferentially along riparian pathways. Genetic results showing related individuals found over 40 km apart imply dispersal distances far greater than the typical home range for muskrat (130 m). These findings suggest that rapid metapopulation recovery is achieved by the long-distance migration of individual muskrat, primarily through riparian corridors in the delta, as well as the passive transport of individuals during large flood events. Results further show the effects of wetland loss on the muskrat metapopulation’s total size and spatial extent. These findings suggest that landscape change is driving a reduction in both the total population size and spatial extent of the muskrat metapopulation of this inland delta.

## Results

### Eruptive dynamics

**Fig. 1:**
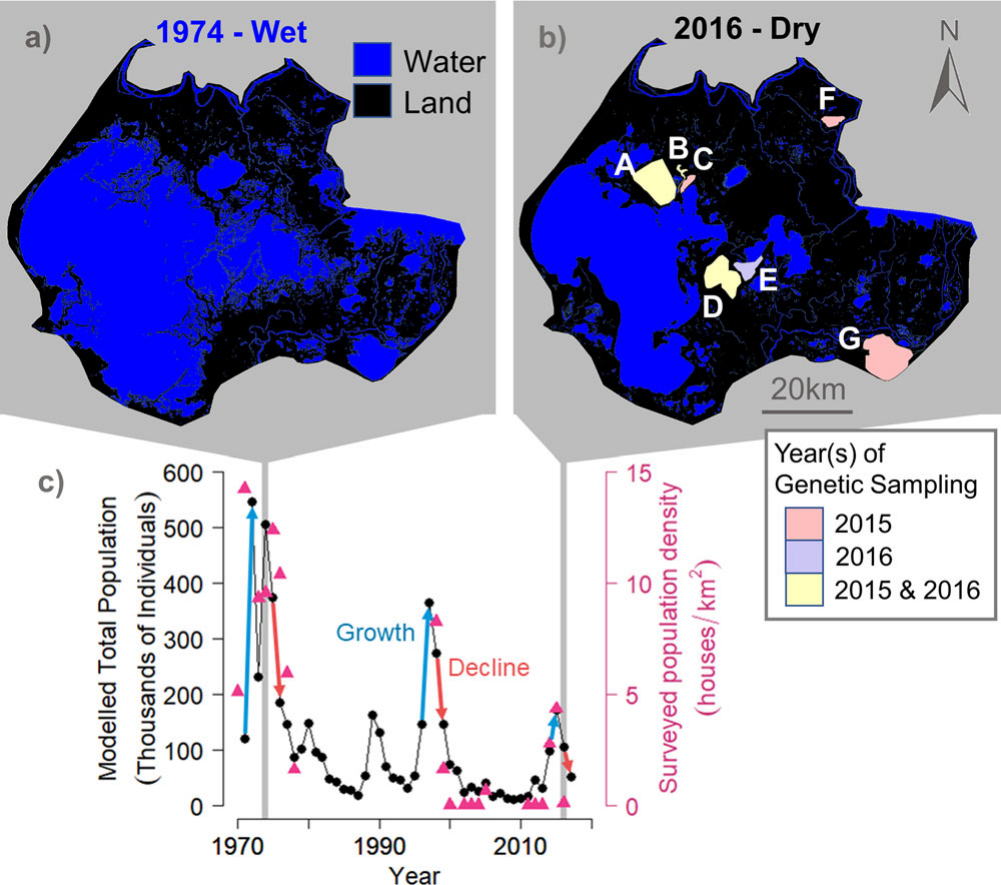
Combined genetic and agent-based modeling analysis of the muskrat population in the Peace-Athabasca Delta. Binary land/water maps of the Peace-Athabasca Delta study site in (**a**), 1974 and (**b**), 2016, showing conditions in wet and dry years, respectively. Polygons in (**b**) indicate sites at which genetic sampling of muskrat took place in 2015 (pink), 2016 (purple), or in both 2015 and 2016 (yellow). **c** The modeled total population and surveyed population density of muskrat in the delta. Agent model results are shown as the median of *n* = 30 realizations of the model. Modeled total population is two times the value of model output and is reported here even though the model simulates females only, based on a study of muskrat in the delta showing nearly even breeding season ratios of males to females^12^. Surveyed population density is estimated as the median density across all surveyed locations in a given year, with the number of locations observed each year varying from *n* = 10 to *n* = 62 sites^10^. Three successive periods of net population growth and decline are indicated in blue and red, respectively, and were selected for source sink mapping in Fig. 3.

Agent-based simulations show that the number of muskrats in the delta is characterized by a series of peak population values followed by sharp declines in the population, concordant with the population survey record (Fig. 1c). Total modeled population size—obtained by doubling model output of the number of females at each time step^12^—ranges from a minimum value of 10,010 individuals in 2009, to a maximum population size of 546,619 in 1972, with values obtained by taking the median across 30 model realizations. Successive modeled periods of eruptive population growth (1971–1972, 1996–1997, 2014–2015) show temporally declining peak population values that reproduce declines in peak population values in the population survey record (Fig. 1c). However, based on genetic analyses, measures of effective population size, N_e,_ using NeEstimator are very low, at 103.7 individuals (95% confidence interval = 88.2–123.5) (6 sites) for 2015 and 61.4 individuals (95% confidence interval = 50.2–76.8) (4 sites) for 2016 (Supplementary Table 1).

Using agent model results, the total modeled population size at four randomly selected sites was examined in 2009, the year with the lowest total modeled population size (Supplementary Fig. 1). Results show sites whose local population has gone extinct (D, B), one site where the local population goes extinct in most but not all realizations of the model (E), and one site whose local population consistently survives, indicating site-dependent local extinction.

### Dispersal behavior and genetic relatedness

**Fig. 2:**
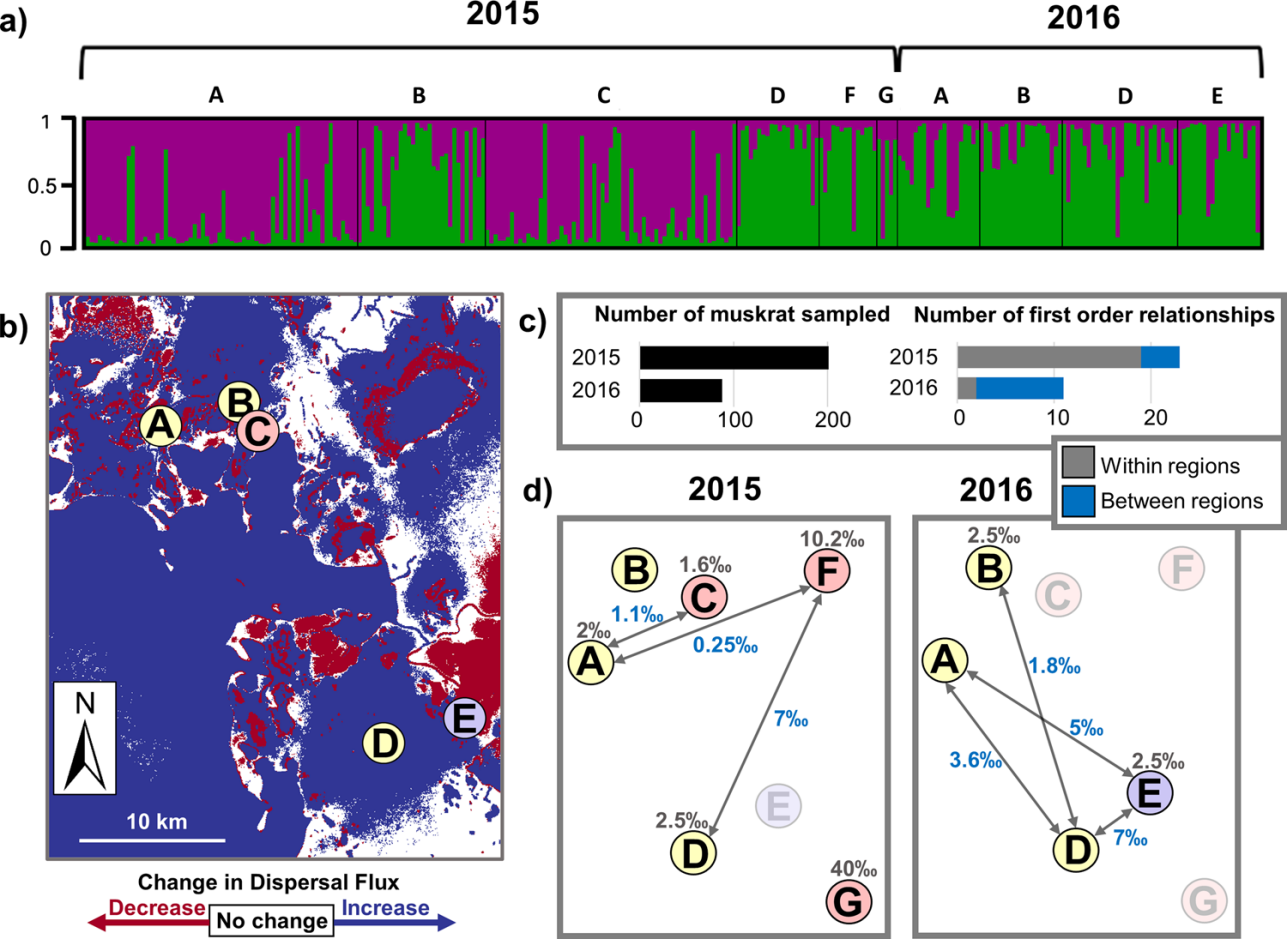
Genetic population structure, agent model dispersal behavior, and genetic relatedness results for muskrat in the delta. **a** STRUCTURE results assign fractions of the ancestry of individual sampled muskrat (shown with vertical bars) to population 1 (purple) or population 2 (green), with individuals ordered by year and sample site. The vertical axis represents the fraction of ancestry from either population, ranging from 0 to 1. **b** Dispersal flux in the agent model, the number of dispersing individual muskrat traveling through a given location, is shown as the difference between dispersal flux in 2016 and 2015 for a portion of the delta encompassing genetic sample sites A through E. The map indicates locations of increased dispersal (blue), locations of decreased dispersal (red), and locations where the amount of dispersal is unchanged across the two years (white). Colored circles show genetic sampling site locations. Colors indicate the year of sampling according to the color scheme in Fig. 1b. Dispersal flux data shown here is the mean of *n* = 30 model realizations. **c** In each year, the number of muskrat sampled and the number of first order relationships. First order relationships are made up of pairs of individuals found within the same sampling site (gray) or pairs of individuals from different sampling sites (blue). **d** The per mil of first order relationships observed out of all possible relationships within (gray) or between (blue, along arrows) sampled sites in each year. Distances between sites are not to scale. Sites not sampled in a given year are grayed out.

Agent simulation results for dispersal show enhanced dispersal flux values in 2016 versus 2015 (Fig. 2b). Although there are some areas of decreased dispersal in 2016 compared to 2015 (red regions in Fig. 2b), we interpret this model output with a wider focus. Namely, that this area of the delta is predominantly experiencing greater dispersal in 2016 than in 2015 (blue regions in Fig. 2b), particularly in the area between sites sampled in 2016 (site A, B, D, and E). In this central region of the delta, this enhanced dispersal is coincident with genetic results showing an increase in the number of related individuals across sample sites and a decrease within sites (Fig. 2c, d). Genetic relatedness results indicate that in 2015, out of the 23 first-order relationships identified, 19 (82.6%) were between individuals at the same site, versus only 2 (18.2%) out of 11 such relationships in 2016. (Fig. 2c, Supplementary Table 2). Coincident with this increase in related individuals across sites, microsatellite data for 2016 relative to 2015 show a decrease in population structure and an increase in homogeneity (Fig. 2a, Supplementary Fig. 2). This trend is corroborated with R_ST_ values showing significant pairwise genetic differences between sites in 2015 but not in 2016 (Supplementary Fig. 3). Cytochrome b analyses show a greater haplotype diversity in 2016 (0.88) than in 2015 (0.67) with a general mixing of haplotypes across all sampled locations (Supplementary Fig. 4).

Agent model simulation results in the des Rochers River region (indicated in Supplementary Fig. 5a) show enhanced dispersal in 2016. Dispersal occurs preferentially through hydrologic features, in this case the des Rochers River (Supplementary Fig. 5b). Results of genetic relatedness analysis, meanwhile, show three related pairs of individuals detected between site F, located in the des Rochers region, and two sites in the central region (Fig. 2d). These results, indicating the presence of related pairs of individuals tens of kilometers apart, suggest that long-distance dispersal by individual muskrats is occurring on the order of tens of kilometers per year. Since muskrat in the delta typically live for two years or less (less than 5% of individuals reach 3 years of age), these distances were likely covered in one or two discrete, long-distance spring dispersal events^12^. This finding is consistent with the model depiction of long-distance migration of individual muskrat through preferential hydrologic pathways.

### Source-sink dynamics

**Fig. 3:**
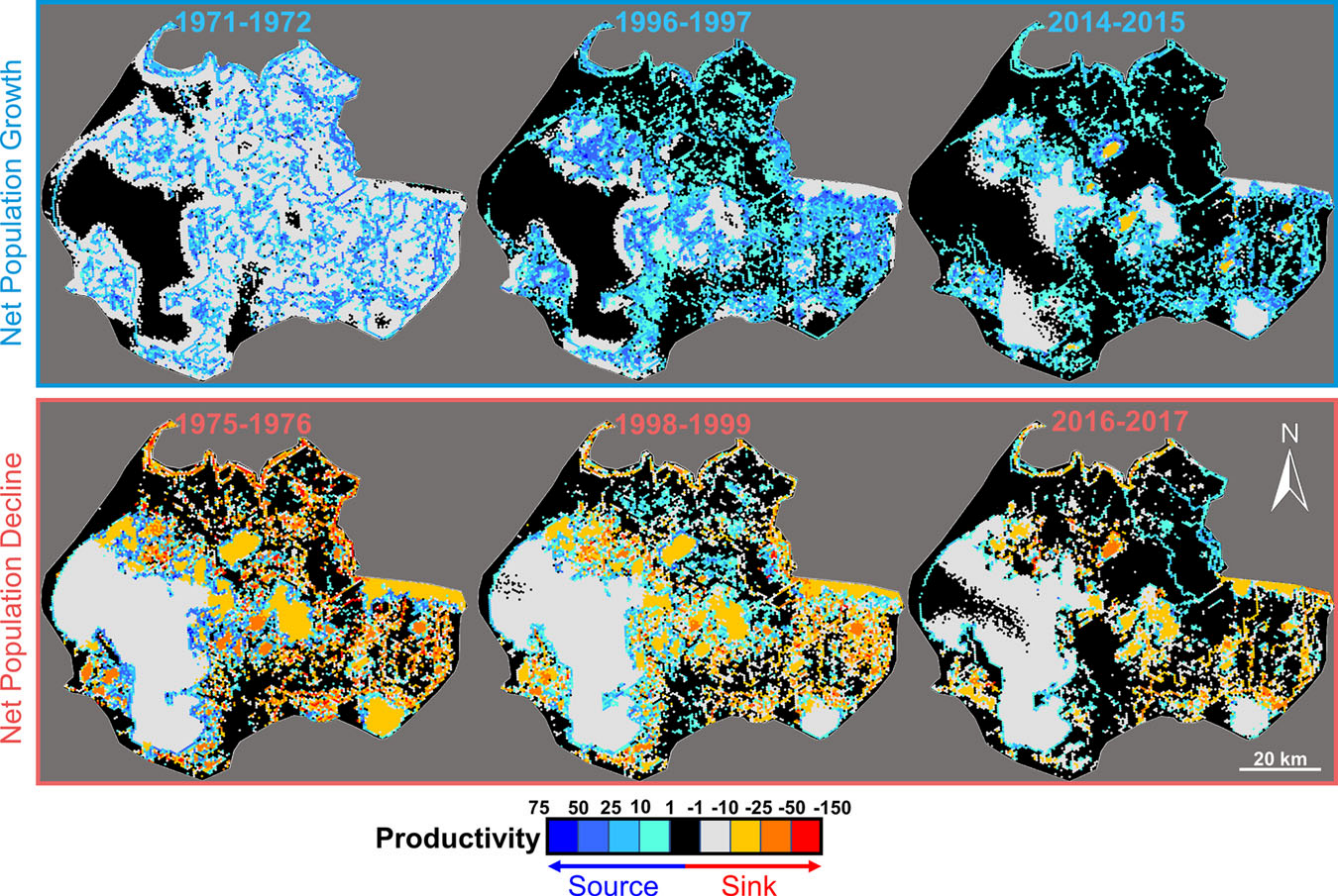
Productivity maps for muskrat in the delta during successive years of net population growth and decline. Maps of productivity (the number of births-deaths of muskrat in each 0.28 km^2^ area) in the delta indicate the locations and strength of sources and sinks in the delta for three successive years of net population growth (1971–1972, 1996–1997, 2014–2015) and subsequent net population decline (1975–1976, 1998–1999, 2016–2017), averaged across *n* = 30 model realizations.

Productivity maps generated from agent model results, showing the number of births minus the number of deaths for muskrat in a given location, indicate locations contributing to net population growth (positive values of productivity) or to net population decline (negative values of productivity)^23^. Results show the delta landscape transitioning on an interannual basis from a state of having many landscape features acting as population sources (years of net population growth in 1971, 1996, and 2014, shown in the top panel of Fig. 3) to the landscape having many population sinks (years of net population decline in 1975, 1998, and 2016, shown in the bottom panel of Fig. 3). These modeled states correspond to eruptive population growth after flooding and post-flood die-offs, respectively. Comparing successive years of net muskrat population increase, the area of the delta serving as a source or sink has decreased from 79% to 55% to 42% in each of the periods 1971–1972, 1996–1997, and 2014–2015, respectively (Fig. 3). This indicates a long-term decline in the area where muskrats are present, whether born or dying in a given location. Comparing the 2014 population increase year to prior years of increase (1971 and 1996), the last year of net population expansion was less productive for muskrat than in previous years. These comparisons also show that in years of net population increase or decrease, multiple locations serve as sources or sinks (Fig. 3).

## Discussion

Agent modeling results show muskrat population dynamics characterized by periods of eruptive population growth followed by die-offs that emerge from the simulated life sequence of up to 546,619 individuals on the floodplain (Fig. 1c). However, microsatellite analyses show extremely low effective population size values (on the order of tens to hundreds of individuals in 2015 and 2016), likely the consequence of repeated die-offs of muskrat resulting in genetic bottlenecking. The agent model and genetic results consistently illustrate a population characterized by rapid eruptive growth and massive die-offs.

We find greater modeled dispersal flux, greater relatedness across sample sites, and less genetic structure in 2016 compared to 2015. Interestingly, 2016 has a smaller modeled population size compared to 2015, with critical habitat and the on-the-ground population density decreasing over the same period^10^. This suggests that muskrat must disperse further under conditions of overcrowding and degraded habitat, with many dying off in the search for suitable habitat.

Our simulations indicate that the delta’s muskrat population is a metapopulation, comprised of many populations from permanent and ephemeral wetlands across the delta. Though local populations—populations of muskrat in subregions of the delta such as the subregions whose populations were tracked in the model (Supplementary Fig. 1b)—may go extinct (=sink) during dry periods, some “seed” populations persist. Following a flood event and ensuing increase in habitat availability and connectivity, the seed (=source) population repopulates sites where habitat has been replenished. While a small number of muskrat likely migrate into the delta during spring dispersal events annually via the rivers that drain into the delta, model results show that local surviving populations of muskrat within the delta numbering in at least the tens of thousands serve as the primary “seed” that disperses and supports rapid population resurgence following flood events. Sites with nonzero local populations thus sustain the delta’s muskrat metapopulation through dry periods between flood events (Fig. 1b, c, Supplementary Fig. 1b).

Simulation and genetic results independently support our conclusion that repopulation requires the occurrence of long-distance dispersal of muskrat in the delta. Both lines of analysis point to long-distance dispersal by muskrat such that newly replenished habitat following flood events results in ensuing eruptive growth (Fig. 1a, c). Genetic results show related muskrat pairs tens of kilometers apart, confirming long-distance dispersal behavior across the floodplain (Fig. 2d). Agent model results illustrate that muskrats use hydrologic features during long-distance migration (Supplementary Fig. 5). We conclude that the mechanism for rapid increases in muskrat abundance from low values after flood waters have subsided is a viable metapopulation that sends successful propagules from “seed” populations to extirpated sites that have been newly replenished. Given the high reproductive rate of muskrat (up to 15 female offspring per mating female per year), even low numbers of arriving females seed local populations at new sites^12^. The metapopulation then contracts with subsequent years of desiccation and negative muskrat herbivory feedbacks, with areas of the delta becoming population sinks and many local populations going extinct. This process is demonstrated on interannual time scales when the landscape transitions from a network of sources in years of net population growth to a network of sinks in years of net population decline, in tandem with individual flood events (Fig. 3 top and bottom panels, respectively). On multidecadal time scales, source-sink mapping shows that the most recent year of net population increase, 2014, was less productive of muskrat than prior eruptive population growth years.

The source-sink networks show a reduction in spatial extent of the species on the floodplain, even in years of peak abundance, due to recent dramatic declines in habitat availability (Fig. 3). This finding is consistent with recent hydro-limnological analysis showing reduced effects of 2014 flooding compared to past events^3^. The multitude of areas of the delta that serve as sources or sinks in any given year of substantial increase or decrease in muskrat numbers highlights that no single portion of the delta is most important for muskrat persistence. These results have a conservation implication: if it becomes necessary, reintroduction of muskrats may be successful in a multitude of water bodies in the delta given their long dispersal range as long as release occurs into a body of water with appropriate vegetation and as long as the water body persists through reproduction.

These findings have larger significance. Given that there are empirical and anecdotal reports of muskrat population declines across North America^24–26^, future work should investigate landscape change and wetland habitat loss as possible drivers of changing muskrat abundance at additional sites across the continent. In addition, the story of muskrats in the delta has broader implications for the delta ecosystem as a whole. Habitat loss, among other drivers, is implicated in the widespread documented decline of North America’s avifauna^27^. Over one hundred bird species, many migrating from across North America, breed at the delta and have similar habitat requirements to the muskrat^28^. While previous research on aquatic birds in the delta has investigated contaminant levels in colonial waterbird eggs, our results suggest that quantifying the effects of landcover change in the delta on waterbird populations would be a fruitful avenue for future studies^29,30^. These species are also likely being adversely affected by the overall decline of freshwater distribution and abundance in the delta, as are hundreds of native plants, fish, insects, and terrestrial animals. Our results have conservation implications for extremely large, remote regions where landscape change is putting local fauna at risk, even in protected ecosystems.

## Methods

### Agent modeling

The agent model for muskrat in the delta was developed using HexSim, an agent-based ecological model that allows for spatially explicit simulation of wildlife population dynamics^31,32^. The HexSim agent model of muskrat incorporated the entire delta in a modeling grid containing 1717 rows of hexagons by 1760 hexagons per row, for a total of 3,021,920 hexagons. Operating on an annual time step, the model tracked up to 273,310 females annually through their life cycles from 1971 to 2017. Given the computational intensity of the model (a runtime of ~16 h per realization), the number of realizations was limited to thirty after examination of model output for the ensemble. Boxplots showed good agreement across model realizations in the timing and magnitude of population peaks, die-offs, and years of low abundance, as well as normally distributed total population size in the majority of years simulated, suggesting that the central tendencies for total population size, dispersal and productivity maps were adequately captured (Supplementary Fig. 1a).

An initial population size for the delta was estimated using an observed muskrat “house” count at a well-studied site, Egg Lake. Records for 1971 show 179 houses, yielding an initial population size of ~448 females at that lake. This estimate was scaled up to a population estimate for the entire delta by accounting for the fraction of critical habitat in the delta occupied by Egg Lake in 1972 (4.88 km^2^ out of 651.77 km^2^) to yield an initial population of 59,701 females for the entire delta.

#### Muskrat movement behavior

The delta model was developed to account for three broad categories of spring movement behaviors for individual muskrat:

##### (i) Local movements during spring dispersal

To represent the spring shuffle within the home ranges of muskrat at their home lake, an “exploration event” allows every individual to search their local surroundings (up to 500 hexagons, or 1.6 km^2^), with the goal of establishing a home range. Individuals that succeed establish a home range and finish the movement event. Individuals that are unsuccessful at establishing a home range as a result of local movement engage in long-range dispersal, described in (ii) below. In the spring, muskrat home ranges typically shuffle within a given water body at the onset of breeding^12,33^. Home range adjustments are typically at the scale of several hundred meters away from previous territory^13^.

##### (ii) Long-range spring dispersal

For individuals that do not successfully establish a home range with local movements in (i), a long-range dispersal event occurs, and it is parametrized based on literature values for muskrat dispersal rates. Based on the highest values of muskrat emigration rates (not attributed to passive transport via flooding) of 60 km/year, we set a dispersal distance of 1000 hexagons, or about 60 km of travel^34^. In addition, such dispersal events are constrained by the fact that muskrat movement is more limited on land than on water. Muskrat are typically observed to move over land on the order of miles^13,33,35^. However, in water they have been observed to travel much further distances irrespective of current; for instance, a single muskrat was observed to travel 50 km “against the current” in 15 days^34^. We therefore infer that higher reported rates of emigration for muskrat are made up primarily of travel through surface water features, combined with an ability of individual muskrat to travel over land up to 3 km.

To represent this in the model, we first used the annual water/shoreline/land maps of the delta to generate annual dispersal maps based on a dispersal metric for particular environment categories. For these maps, water and shoreline pixels received a score of 10, and land pixels received a score of zero. This yielded dispersal maps whose hexagons have values of zero when they entirely overlie land pixels, 10 when they entirely overlie water pixels, and values in the range (0,10) for shoreline regions. Then, at each step of muskrat travel along its dispersal path, the difference of the hexagon score from 10 is evaluated and added to that individual’s dispersal penalty. Land hexagons therefore have a resistance of 10, and water hexagons a resistance of 0, with shoreline regions incurring an intermediate resistance between 0 and 10. The resistance values of encountered hexagons are tracked cumulatively for each individual while it disperses. When an individual reaches a resistance threshold of 500, the individual must stop dispersing. This resistance threshold of 500 is equivalent to 3 km of overland travel. So, an individual dispersing with a path entirely over land can go 3 km per year from their prior home range, but if their dispersal is entirely through water, then there is a travel limit of 60 km in a year.

During long-range spring dispersal, individuals follow a constrained random walk to find a suitable place to settle. When selecting the adjacent hexagon to explore, individuals prefer hexagons with values between 2 and 10 (shoreline and water hexagons) at the expense of hexagons with values between 0 and 1 (land or mostly land hexagons), and they are influenced by their prior direction of travel with autocorrelation of 50%. At the completion of their long-range dispersal, individuals repeat the local movement exploration event to search for a suitable location to settle within their newly discovered home range. Individuals that do not succeed are removed from the simulation, representing death because they did not successfully establish a home range after long-range dispersal and succumbed to predation or starvation, or representing that they have migrated out of the delta.

##### (iii) Enhanced dispersal due to flooding

In years of known, large-scale flooding in the delta (1972, 1974, 1996, 1997 and 2014), a flood dispersal event is applied to simulate the effects of flooding on muskrat dispersal. A dispersal map is applied in which all hexagons in the delta have a value of 10, such that there is no resistance penalty for movement (a resistance value of 0) and the resistance threshold described in (ii) is never reached. When determining the range of distances for dispersal of muskrat due to floodwaters, we drew on literature values. While some muskrat remain in the water and disperse during flooding, yielding emigration rates of up to 120 km/year, others find refuge in trees or on rafts that are swept into trees and move no further^34,36,37^. To represent this range of outcomes, the distribution of path lengths was assigned a log-normal distribution, with a mode of 10 hexagons (600 m) and a median of 100 hexagons (6 km). Due to the ability of muskrat to swim up-current over tens of kilometers, this log-normal distribution functions independently of current^34^. This yields a distribution in which half of affected muskrat remain within six kilometers of their home ranges, while others may move tens of kilometers away. After the flood-induced dispersal movement event is complete, individuals undertake an exploration event as defined in *(i)* using the habitat map for that year, which represents the habitat available for home range establishment after floodwaters have receded.

*Additional parameters for the Dispersal event are:.* Repulsion from hexagons with values between 0 and 1 (land or mostly land hexagons); Attraction to hexagons with values between 2 and 10 (shoreline and water hexagons), with a Multiplier of 5; and Percent Auto-Correlation of 50% with a Trend Period of 3 hexagons.

#### Source-sink mapping

Model output was mapped to evaluate the spatial distribution of sources, areas of high quality habitat serving as net contributors to the total muskrat population in the delta, and sinks, areas of low quality habitat serving as net detractors from the total muskrat population in the delta^38^. Mapping population dynamics in this way allows us to visualize the population dynamic effects of a spatially heterogeneous landscape. The location and intensity of sources and sinks were mapped at selected years to test our hypothesis that the delta’s flood regime drives interannual changes in the spatial distribution of source-sink dynamics of the muskrat metapopulation.

Productivity, defined as the total number of births minus deaths in each area, was used as a simple measure of source and sink quality on the landscape (Fig. 3)^39^. We mapped productivity across the delta for three pairs of years, each associated with a population increase following a flood and subsequent die-off: (1971–1972) and (1975–1976), (1996–1997) and (1998–1999), (2014–2015) and (2016–2017) (Fig. 3). The years were selected based on results of realizations from thirty model simulations (Fig. 1c). Maps show the source or sink ensemble average values over those thirty modeled realizations.

Source-sink mapping was carried out in HexSim using a set of simulation processes: the patch map, individual locations updater function, and productivity report modeling framework tools, as well as the build hexmap hexagons, clip hexmap, renumber patches, and map productivity report utilities developed by Nathan Schumaker^40^. Once in each year of the simulation, the model’s muskrat population was sampled within areas of regular tessellations comprised of hexagonally shaped areas with radii of 5 hexagons each. This sampling was executed in the model by recording birth and death statistics within each area.

#### Dispersal flux mapping

Dispersal flux, the number of individuals passing through a given location per year, was mapped as the difference in values for the two years in which genetics data were collected, 2015 and 2016 (Fig. 2b). This was done by first exporting hexagon-based dispersal flux tallies for all thirty realizations in the years 2015 and 2016. Then, the mean value of dispersal flux across all 30 realizations was calculated to produce a single average dispersal flux map for each year. Finally, the difference between these two maps was calculated to yield the difference map showing locations of increased, decreased, or unchanged dispersal flux shown in Fig. 2b.

### Genetic analysis

#### Sample collection

Muskrat tissue samples for this study consisted of <2 mm diameter pieces of tail tissue donated by trappers from muskrats trapped for fur and meat during the 2015 and 2016 trapping seasons (November to May). All trapping resulting in donated samples was done so legally under all necessary permits or by individuals with Indigenous trapping rights in the delta as recognized by Treaty 8. All muskrats were collected from the Peace-Athabasca Delta and the lake or creek of collection was noted by the trappers. A total of 200 muskrats were collected from 6 sites in the 2015 trapping season and 88 muskrats were collected from 4 sites in the 2016 trapping season (Fig. 1b, Supplementary Fig. 6). This study was designed to capitalize upon muskrat trapping that was already taking place in the delta, thus, we had no control over the number of samples donated nor the specific location within the delta where samples were collected.

#### DNA extraction and amplification

DNA was extracted from tissue samples using the DNeasy Blood and Tissue kit following the manufacturer’s protocol (Qiagen, Valencia, CA). Partial mitochondrial cytochrome b sequences of 872 base pairs were generated from samples using the same primers described in Mychajliw and Harrison (2014): OzbFW 5ʹCACTCATTCATCGACCTCCCAAC3ʹ; OzbREV 5ʹTGGG- TATGAAGATAATGATAATGGCAAAGTA3ʹ^41^.

Amplifications were carried out in a total volume of 10 µl made up of the following mixture: 1 µl DNA template, 5 µl GoTaq® G2 Hot Start Colorless Master Mix (Promega), 1 µl 5 µM of each primer, 2 µl water. PCR amplifications were performed in an Eppendorf Mastercycler Pro thermal cycler, with an initial denaturation at 95 °C for 15 min, followed by 30 cycles of 30 s of denaturation at 94 °C, 30 s of annealing at 57 °C, and 1 min of extension at 72 °C and a final hold at 4 °C. Negative controls were used in all extractions and PCRs as quality controls. The amplified products were electrophoresed in 2% agarose gels (200 Volts, 20 min), the DNA bands were visualized using SYBR Safe DNA Gel Stain (ThermoFisher Scientific) under UV light and product size was determined in relation to a 100 bp DNA size standard (ThermoFisher Scientific). PCR products were cleaned using ExoSAP-IT (ThermoFisher Scientific) and sequenced by Elim Biopharmaceuticals, Inc. (Hayward, CA). Sequences were aligned using Geneious 7.1.4.

Nine autosomal microsatellite loci (Oz06, Oz08, Oz16, Oz27, Oz32, Oz34, Oz41, Oz43, Oz44) were PCR amplified using primers from Laurence et al. in a total of 288 samples, 200 from 2015 and all 88 from 2016^14^. Amplification was carried out in a total volume of 5 µl made up of the following mixture: 0.5 µl DNA template, 2.5 µl GoTaq® G2 Hot Start Colorless Master Mix (Promega), 0.5 µl of each primer mixture (containing 2 µM of each primer), 1.5 µl water. PCR amplifications were performed in an Eppendorf Mastercycler Pro thermal cycler, with an initial denaturation at 95 °C for 15 min, followed by 30 cycles of 30 s of denaturation at 94 °C, 30 s of annealing at 60 °C, and 1 min of extension at 72 °C and a final hold at 4 °C.

Microsatellite PCR product was sent to the Protein and Nucleic Acid Facility at Stanford University to conduct multiplexed fragment size analysis using an ABI 3130xl Genetic Analyzer. Fragment analysis was done with PCR products for four microsatellite loci (Oz06, Oz27, Oz32 and Oz43) multiplexed in one run and the other five microsatellite loci (Oz08, Oz16, Oz34, Oz41, Oz44) multiplexed in a second run.

### Statistics and reproducibility

#### Agent-based model

Statistics within the model were carried out using HexSim Version 4.0.13.0 (available at www.hexsim.net) using files with code specific to the muskrat simulation to generate model output files, all available at 10.17605/OSF.IO/CHNR6^31,32^. Statistics on model output to calculate mean and median values and boxplots on model output for n = 30 realizations reported in the paper were carried out using R/v.3.4.3^42^.

#### Cytochrome b

To determine how many samples to sequence to capture all cytochrome b haplotype diversity, we plotted haplotype accumulation curves using the package Spider in R with random ordering of samples and 10,000 permutations^42,43^. Based on accumulations curves, we sequenced 150 samples from 2015 and all 87 samples from 2016; one sample from 2016 did not amplify (Supplementary Fig. 7).

#### Microsatellite calling

For all microsatellite loci, samples were PCR amplified, fragment analyzed and scored twice to assure genotyping accuracy. All scoring was conducted in Geneious v7.1.4. Three samples from 2015 were removed due to missing data and/or discrepancies between the two genotyping runs. In our final dataset, 90.3% of alleles from 2015 were confirmed by two identical genotypes and 96.5% of alleles from 2016 were confirmed by two identical genotypes. The remainder were alleles that were successfully captured in only one fragment analysis and were missed in the other run due to allelic dropout. The final microsatellite dataset used for all analyses consisted of *n* = 197 for 2015 and *n* = 88 for 2016 and is provided as Supplementary Data 1.

#### Microsatellite analyses

We investigated the presence of null alleles with a 95% confidence interval using MICROCHECKER v2.2.3 and estimated the frequency of null alleles in each sampling site using genepop v1.0.5 in R^42,44,45^. We also tested for null alleles in the dataset for each year considered as one population. Null alleles were indicated at numerous loci (Supplementary Table 3); however, there was no consistency in loci displaying null alleles across sampling sites and thus all alleles were kept in the analyses.

We calculated the number of alleles and allelic richness (corrected for sample size) for each sampling site in each year using FSTAT V2.9.3.2^46^. We calculated the observed (H_O_) and expected (H_E_) heterozygosity and deviations from Hardy-Weinberg equilibrium using Arlequin 3.5.2.2^20^. When identifying significant deviations from Hardy-Weinberg equilibrium we corrected for multiple comparisons by applying the Benjamini & Hochberg method in R to control for false discovery rate^47^.

We assessed pairwise genetic difference between sites with 14 or more samples (excluding G) by calculating R_ST_ in Arlequin. R_ST_ is similar to F_ST_ but takes allele size into account and is considered to be more suitable for the high mutation rate of microsatellite data^48^.

#### Structure

Population structure was assessed using the Bayesian clustering approach implemented in the program STRUCTURE v2.3.4 run using the admixture model with correlated allele frequencies, 100,000 burn in, and 1 million MCMC iterations^19^. These are the same priors used in previous studies to successfully identify population structure in muskrat using the same microsatellite markers used here^41,49^. We ran 20 independent runs for K from 1 to 10. STRUCTURE was run on a Linux machine and parallelized using the program StrAuto^50^. We used the Evanno method to identify the most probable K as the one with the largest Delta K value^51^. The rate of change in the log probability of the data between successive K values (Delta K) was calculated and visualized using STRUCTURE HARVESTER Web v0.6.94 and structure results were visualized using CLUMPAK^52,53^. We conducted these structure analyses on each year separately as well as both years together (Supplementary Fig. 2). In all STRUCTURE runs, no prior information on sampling location or sampling year is included. The Evanno method indicated that two populations (K = 2, as shown in Fig. 2a), was the most probable (Supplementary Fig. 2c).

#### Estimation of relatedness

We identified putative first-order relatives (parent-offspring or full siblings) by cross referencing output from three different programs—Colony v 2.0.6.5, ML-Relate, and Coancestry^16–18^. We ran Colony assuming polygamy in both males and females and assuming a genotyping error rate of 0.05^12^. We only considered first-order relatives with a probability of 95% or higher. We further verified relatedness by only keeping relationships that were also supported by ML-Relate with a maximum likelihood estimate of relatedness of 0.495 or higher and by Coancestry with a TrioML 95% confidence interval overlapping 0.5 but not 0.125. In Coancestry, we used the triadic likelihood estimator (TrioML) which uses a triad of individuals for estimating pairwise relatedness^54^.

#### Effective population size

Effective population size (N_e_) with 95% confidence intervals using the parametric method was estimated using the linkage disequilibrium method in NeEstimator^15,55,56^. We ran NeEstimator using a random mating system and considered results when excluding alleles with frequencies less than 0.01. This estimation of N_e_ represents the effective population size of the parental generation of the samples. We also estimated N_e_ with 95% confidence intervals using the moment and likelihood methods implemented in MLNe^57^. We ran MLNe without assuming drift-migration equilibrium, set a maximum N_e_ value of 35750 (maximum based on computing limitations), and used both timepoints (2015 and 2016) to estimate N_e_ for both breeding seasons. These two methods of estimating N_e_ have been found to be the most accurate when considering numerous N_e_ estimation methods^58^.

### Reporting summary

Further information on research design is available in the Nature Research Reporting Summary linked to this article.

## Supplementary information

Supplementary Information

Description of Supplementary Files

Supplementary Data 1

Reporting Summary

## Data Availability

The agent-based model and modeling results, including raw data associated with Figs. 1, 2 and 3, are available at 10.17605/OSF.IO/CHNR6. All cytochrome b sequence data have been submitted to the GenBank database under accession numbers MT215718 - MT215954. Figure 2 has associated raw data from the genetics analyses; that microsatellite dataset is included as Supplementary Data 1. Muskrat tissue samples are available from E.A. Hadly upon reasonable request.

## Code availability

Custom code developed for the muskrat agent-based model in HexSim Version 4.0.13.0 is available at 10.17605/OSF.IO/CHNR6.
