# Supplementary material for: Muskrats as a bellwether of a drying delta
Source: Commun Biol. 2021 Jun 24;4:750. doi: 10.1038/s42003-021-02288-7 (PMC8225612; doi:10.1038/s42003-021-02288-7)
Supplement: Supplementary file 1 — Supplementary Information [file 42003_2021_2288_MOESM1_ESM.pdf]

Supplementary Information: Muskrats as a bellwether of a drying delta

Ellen M. Ward[1*†] and Katherine A. Solari[2*], Amruta Varudkar[2], Steven M. Gorelick[1], Elizabeth A. Hadly[2]

[1] Department of Earth System Science, Stanford University, Stanford, CA, USA
[2] Department of Biology, Stanford University, Stanford, CA, USA
† Corresponding author (email: emward@alumni.stanford.edu)

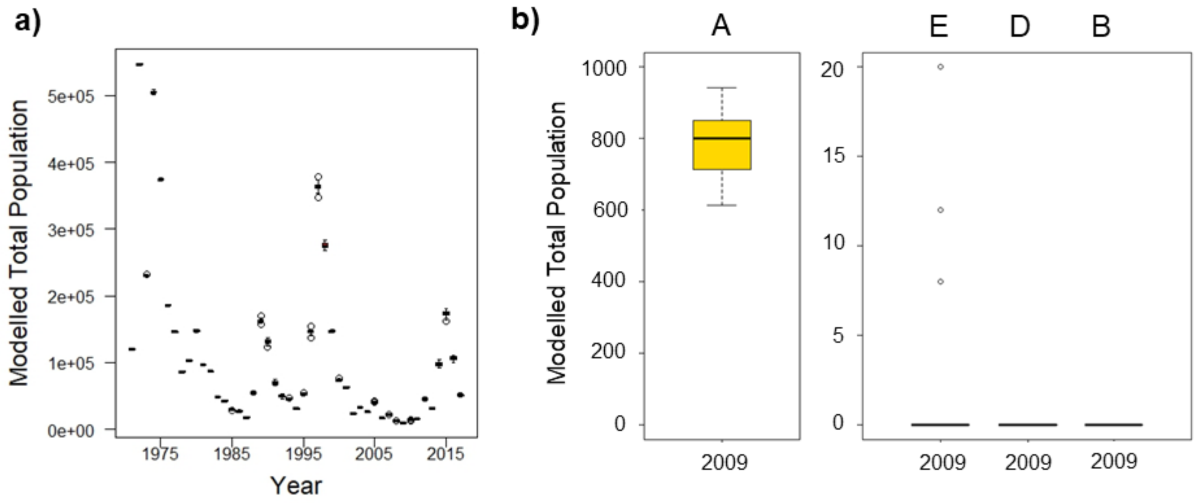

Supplementary Figure 1. Boxplots for the ensemble of 30 realizations. a, Total population is presented as a boxplot across 30 realizations of the model. The distribution of total population size at each time step passes the Shapiro-Wilkes normality test in all but six years (1973, 1991, 1993, 2000, 2005 and 2007) of 46 years total. **b**, Boxplots for modelled total population size at four genetic sampling sites (A, E, D and B, sites shown in Figure 1b) across 30 realizations of the model in 2009, the year with the lowest modelled total population size in the delta. For the boxplots in both a and b, the position of the top whisker is the data point furthest from the top box edge (Q3) that does not exceed 1.5 times the interquartile range (IQR) from the Q3 value. The position of the bottom whisker is the data point furthest from the bottom box edge (Q1) that does not exceed 1.5*IQR below the Q1 value.

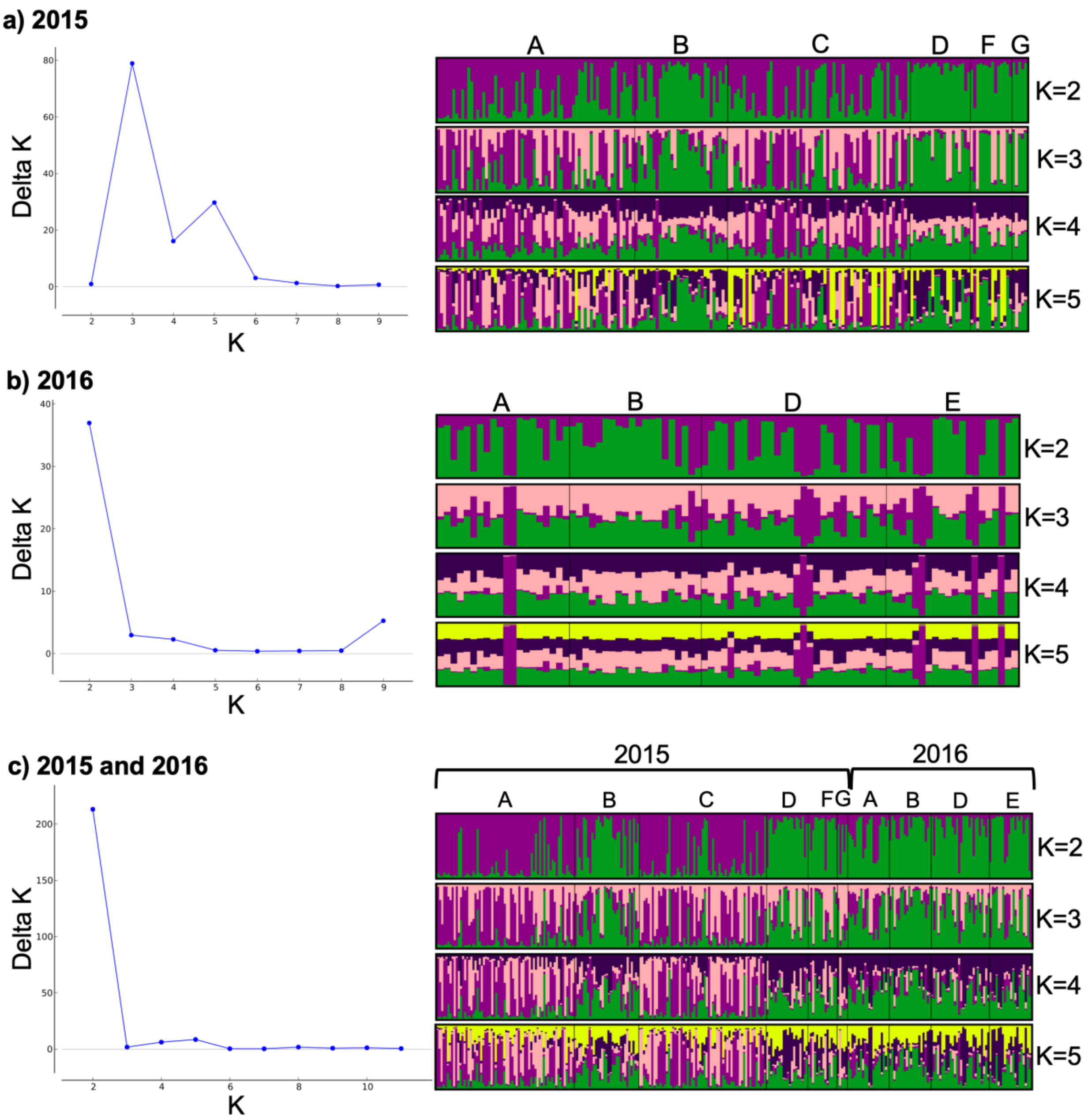

Supplementary Figure 2. STRUCTURE output for 2015 samples in **a**, 2016 samples in **b,** and both years together in **c**. On the left is the ΔK plot for each set of data and on the right is the assignment of individuals to clusters for K=2 to K=5. Each vertical line represents an individual. In **a** and **b,** individuals are grouped by sample site with sites ordered from West to East. In **c**, individuals are grouped by year and then by sample site with sites ordered from West to East. The vertical axis represents percentage of ancestry in each cluster.

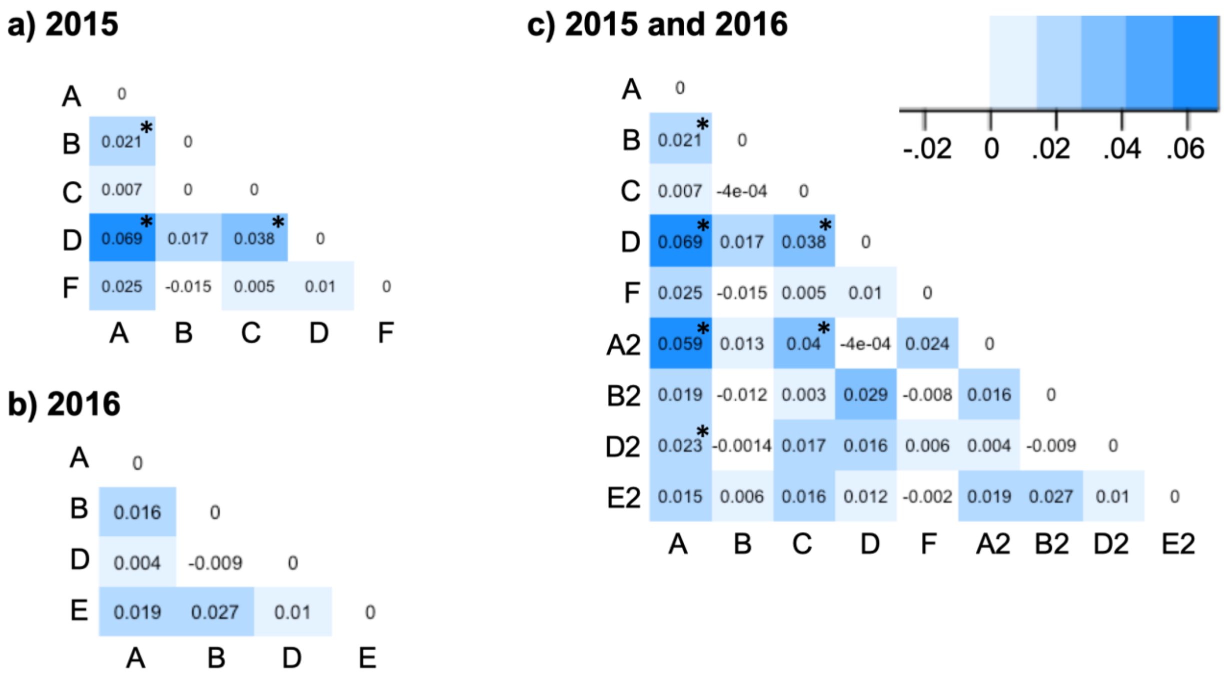

Supplementary Figure 3. Pairwise R_{ST} between sites sampled in 2015 in **a**, 2016 in **b**, and both years together in **c**. In c, sites sampled in 2016 are denoted with a "2" after the site ID. *$p<0.05$. Exact p-values rounded to 4 decimal places for all significant values: 2015 A-B p= 0.0166, 2015 A-D p= 0.0001, 2015 C-D p= 0.0195, 2015/2016 A-B p = 0.0147, 2015/2016 A-D p= 0.0001, 2015/2016 A-A2 p= 0.0001, 2015/2016 A-D2 p= 0.0254, 2015/2016 C-D p= 0.0176, 2015/2016 C-A2 p= 0.0098.

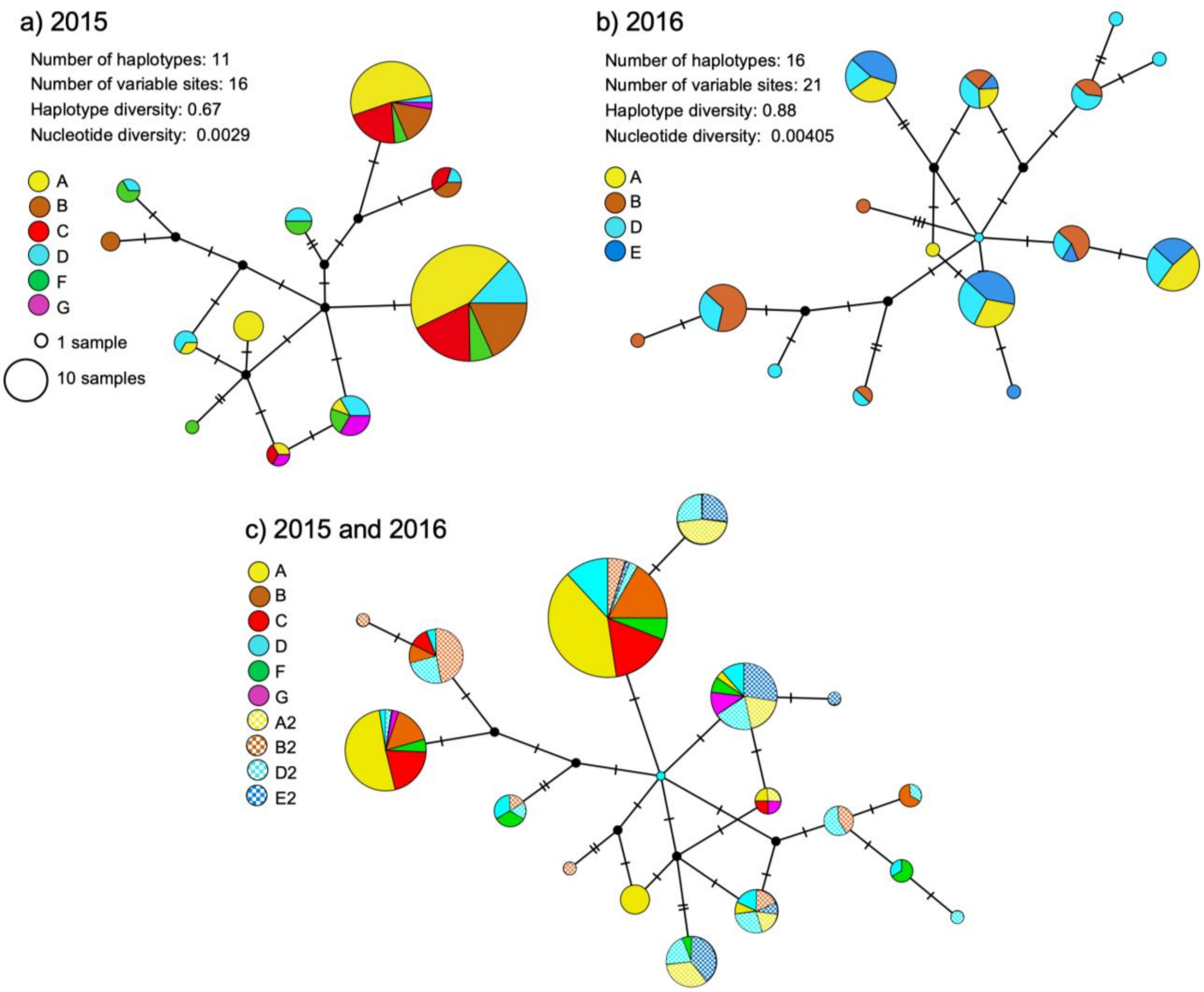

Supplementary Figure 4. Haplotype networks using 872 bp of cytochrome b for 2015 in **a**, 2016 in **b** and samples from both years together in **c**. Each hatch mark indicates a nucleotide difference. In **c**, 2015 samples are shown in solid color and 2016 samples are in checkerboard.

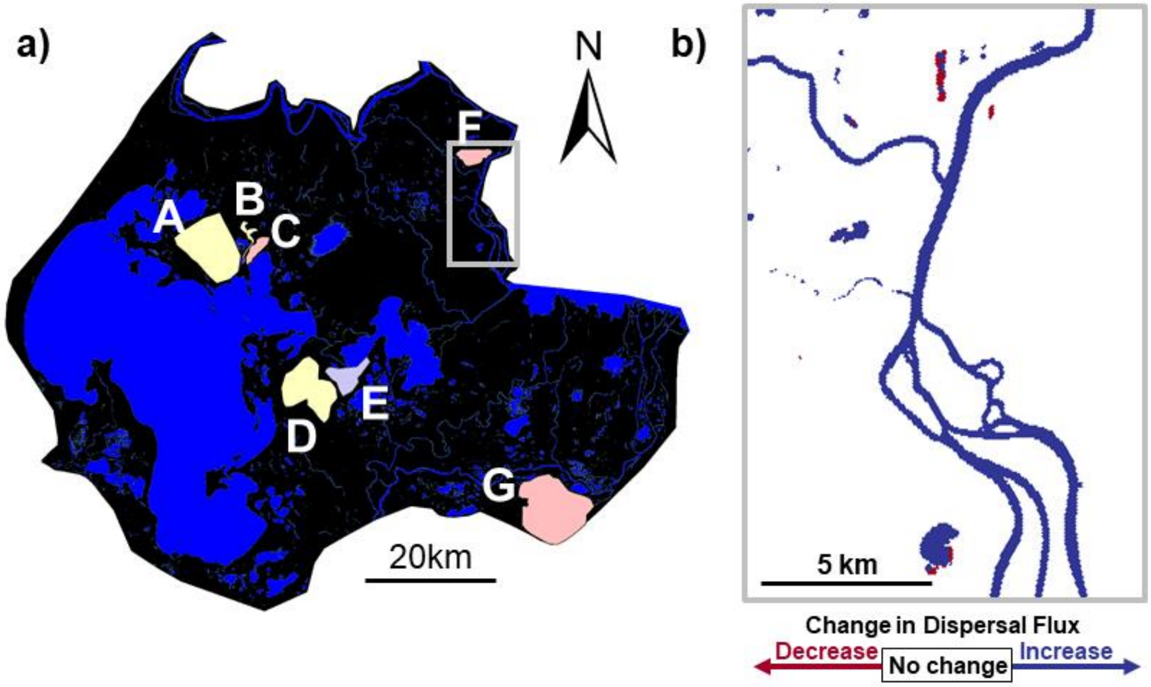

Supplementary Figure 5. Muskrat dispersal behavior in the agent model. **a**, The grey box in the upper-right portion of the binary water/land map of the delta indicates the region mapped in b. Polygons indicate sites at which genetic sampling of muskrat took place in 2015 (pink), 2016 (purple), or in both 2015 and 2016 (yellow). **b**, Dispersal flux in the agent model, the number of dispersing individual muskrat travelling through a location, is shown as the difference between dispersal flux in 2016 and 2015 for a portion of the delta around the des Rochers River. The map indicates locations of increased dispersal (blue), locations of decreased dispersal (red), and locations where the amount of dispersal is unchanged across the two years (white). Dispersal flux data shown here is the mean of 30 model realizations.

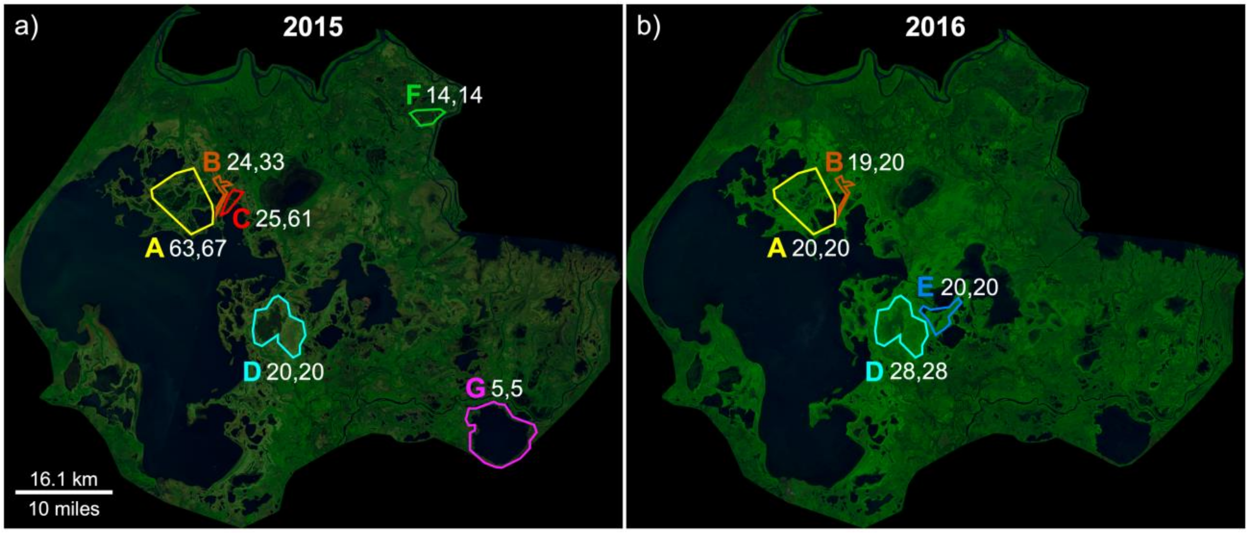

Supplementary Figure 6. Sample sites for each collection year. Sites sampled in 2015 are shown in **a** and sites sampled in 2016 are shown in **b**. The number of samples included in cytochrome b analysis, followed by the number of samples included in microsatellite analysis, are indicated next to each site. Satellite images are from June 2015 and 2016, respectively.

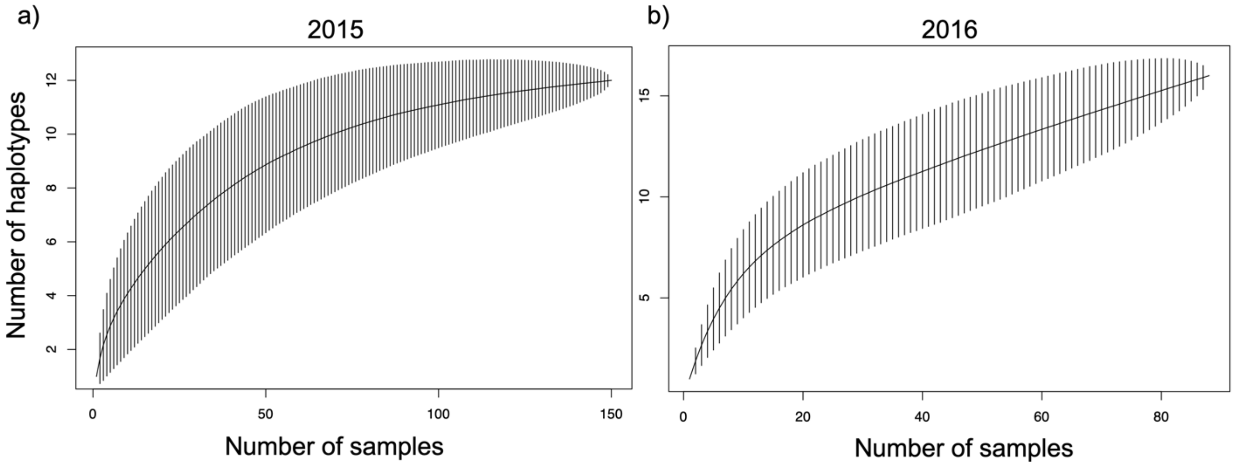

Supplementary Figure 7. Haplotype accumulation curves based on 872bp of cytochrome b for 2015 samples in **a** and 2016 in **b**. Error bars represent one standard deviation.

Supplementary Table 1. Effective population size estimates for each sampling year with 95% confidence intervals.

Year	NeEstimator (0.01 min frequency)	MLNe (likelihood estimate)	MLNe (moment estimate)
**2015**	103.7 (88.2 – 123.5)	235.8 (88.7 – infinity)	infinity
**2016**	61.4 (50.2 – 76.8)		

Supplementary Table 2. 2015 pairwise relatedness estimates followed by 2016 pairwise relatedness estimates when assessing each year separately.

Individual 1	Individual 2	Probability of first order relation (Colony)	Relatedness (ML-Relate)	95% CI (Coancestry)	
**2015**					
A_503	A_518	0.999	0.910	0.741	1
A_610	A_814	0.999	0.919	0.713	1
D_111	F_407	0.999	0.839	0.609	1
A_603	A_816	0.998	0.667	0.303	1
A_510	A_813	0.996	0.717	0.349	1
A _605	A_611	0.996	0.762	0.557	0.931
A_715	C_1409	0.995	0.819	0.601	1
C_201	C_1406	0.995	0.628	0.301	1
C_1313	C_1408	0.995	0.624	0.308	0.884
A_510	A_802	0.993	0.747	0.427	1
D_110	D_118	0.987	0.728	0.359	1
A_505	A_812	0.984	0.715	0.500	0.889
C_208	C_1309	0.984	0.679	0.500	0.845
F_408	F_411	0.983	0.614	0.371	0.926
A_612	A_712	0.982	0.674	0.176	0.844
C_201	C_1209	0.980	0.724	0.500	0.932
D_113	F_403	0.979	0.495	0.152	0.833
G_1504	G_1505	0.976	0.607	0.285	0.911
F_403	F_408	0.975	0.496	0.247	0.831
A_706	F_409	0.966	0.666	0.493	0.931
C_206	C_1404	0.965	0.634	0.269	0.829
A_710	A_817	0.953	0.615	0.143	0.919
C_1209	C_1309	0.950	0.646	0.292	0.804
**2016**					
D2_2705	E2_2503	1	0.920	0.707	1
B2_2807	D2_1903	0.999	0.861	0.528	1
A2_2012	D2_1916	0.995	0.757	0.524	0.927
A2_2006	E2_2513	0.994	0.754	0.500	0.924
D2_1916	E2_2506	0.993	0.659	0.349	0.922
D2_2703	E2_2512	0.983	0.610	0.313	0.861
E2_2502	E2_2503	0.983	0.678	0.367	0.881
D2_2705	E2_2502	0.982	0.678	0.283	0.881
A2_2008	D2_2701	0.977	0.711	0.500	1
A2_2001	A2_2009	0.971	0.670	0.500	0.896
A2_2011	E2_2518	0.965	0.684	0.286	0.925

Individuals are colored by which lake they are from. Relatedness from MLrelate is the maximum likelihood estimate of pairwise relatedness. 95% confidence intervals for Coancestry analyses are for triadic likelihood estimator (TrioML).

Supplementary Table 3. Summary statistics for microsatellite data

	2015							2016				
Sampling site	A	B	C	D	F	G	Total	A2	B2	D2	E2	Total
N	66	31	61	20	14	5	197	20	20	28	20	88
h	6	4	4	7	6	3	11	5	7	12	6	16
Oz06 (N_a)	4	5	3	5	4	4	5	4	3	5	5	6
Null allele	**0.194**	0	0.045	0.165	0	-	**0.111**	0.021	0.084	0	**0.240**	**0.100**
r	2.5	2.73	2.39	3.09	2.93	4	2.65	4	3	4.71	5	5.05
H_O	0.3	0.45	0.43	0.5	0.57	0.4	0.4	0.55	0.2	0.57	0.35	0.43
H_E	0.47*	0.43	0.44	0.52	0.54	0.53	0.46	0.6	0.34	0.59	0.73*	0.60*
Oz32 (N_a)	12	12	11	10	7	6	17	11	12	11	10	13
Null allele	0.004	0.031	0	**0.090**	0.0179	-	0.023	0	0	0.008	0.024	0.014
r	6.36	6.95	6.19	6.13	5.47	6	6.69	11	12	10.62	10	10.54
H_O	0.89	0.9	0.89	0.7	0.79	1	0.87	0.9	0.9	0.86	0.8	0.86
H_E	0.89	0.92	0.88	0.88	0.86	0.89	0.90*	0.89	0.91	0.9	0.89*	0.89
Oz43 (N_a)	15	14	16	11	10	5	17	12	11	11	11	15
Null allele	**0.033**	0	0	0	0	-	0.011	0.042	-	-	**0.1**	0.0359
r	6.79	6.91	6.4	6.14	5.83	5	6.79	12	11	10.32	11	11.49
H_O	0.82	0.97	0.87	0.85	1	0.6	0.87	0.8	0.85	0.93	0.65	0.82
H_E	0.9	0.91	0.88	0.87	0.85	0.8	0.9	0.88	0.88	0.88	0.86	0.87*
Oz27 (N_a)	4	5	4	5	5	4	6	4	6	5	4	7
Null allele	0.0286	0	0	0.0504	0.071	-	0.03	0.060	0	0.005	0	0.006
r	3.06	3.41	3.1	3.88	3.81	4	3.35	4	6	4.64	4	4.73
H_O	0.47	0.68	0.56	0.65	0.5	0.6	0.55	0.5	0.6	0.64	0.65	0.6
H_E	0.52	0.64	0.53	0.72	0.67	0.64	0.59	0.66	0.66	0.62	0.57	0.63
Oz41 (N_a)	10	8	10	9	6	4	10	8	7	9	9	11
Null allele	0.011	0.121	0.112	0.162	0.189	-	0.099	0.105	0	0.083	0.071	0.070
r	5.58	5.06	5.59	5.21	5.11	4	5.49	8	7	8.13	9	8.15
H_O	0.92	0.84	0.84	0.8	0.64	0.8	0.85	0.7	0.8	0.71	0.8	0.75
H_E	0.84	0.8	0.86	0.82	0.83	0.78	0.84	0.72	0.74	0.72	0.81	0.74
Oz16 (N_a)	10	9	8	6	6	5	12	7	8	9	7	11
Null allele	0	0	0.029	0	0	-	0.008	0.088	0	**0.098**	0.072	**0.061**
r	4.78	5.34	4.49	4.31	4.95	5	4.89	7	8	7.78	7	7.37
H_O	0.76	0.87	0.75	0.75	0.86	0.6	0.79	0.6	0.9	0.57	0.65	0.67
H_E	0.78	0.83	0.78	0.78	0.86	0.76	0.8	0.81	0.83	0.78	0.83	0.81
Oz08 (N_a)	2	2	2	2	1	1	2	1	1	2	1	2
Null allele	0	0	0	0	NA	-	0	NA	NA	0.193	NA	0.107
r	1.08	1.3	1.41	1.25	1	1	1.23	1	1	2	1	1.65
H_O	0.02	0.07	0.1	0.05	0		0.05			0.07		0.02
H_E	0.07*	0.06	0.13	0.05	0.25 *		0.05			0.20*		0.05
Oz44 (N_a)	11	7	12	6	5	3	12	8	6	9	8	11
Null allele	0	0	0	0.070	0.090	0	0	0	0.03	0.051	0.116	0.065
r	4.04	4.14	4.52	3.87	4.27	3	4.32	8	6	7.98	8	7.87
H_O	0.7	0.65	0.72	0.55	0.57	0.6	0.67	0.95	0.55	0.64	0.8	0.73
H_E	0.63	0.68	0.7	0.67	0.78	0.51	0.68	0.76	0.65	0.72	0.83	0.75
Oz34 (N_a)	8	8	8	7	6	4	8	6	6	6	6	7
Null allele	0.138	0	0.013	0.001	0	-	0.070	0	0	0.054	0	0
r	5.05	5.19	4.85	4.97	4.51	4	5.3	6	6	5.92	6	6.34
H_O	0.89	0.9	0.82	0.85	0.71	0.6	0.85	0.85	0.9	0.71	0.9	0.83
H_E	0.82	0.83	0.8	0.83	0.81	0.64	0.84	0.80*	0.81	0.78	0.79	0.8

Null allele: null allele frequency estimates as calculated in genepop, N: number of samples, h: number of cytochrome b haplotypes, N_a: Number of microsatellite alleles, r: allelic richness (corrected for a minimum sample size of 5 for 2015 and 20 for 2016), H_O: Observed

heterozygosity, H_E: Expected heterozygosity, *: Deviations from HWE at 95% confidence (corrected with FDR). The null allele frequency estimate for each year when all samples are considered together is given in the "Total" column. Loci that were identifies by MICRO-CHECKER as null alleles are in bold.
