# Supplementary material for: Muskrats as a bellwether of a drying delta
Source: Commun Biol. 2021 Jun 24;4:750. doi: 10.1038/s42003-021-02288-7 (PMC8225612; doi:10.1038/s42003-021-02288-7)
Supplement: Supplementary file 2 — Description of Supplementary Files [file 42003_2021_2288_MOESM2_ESM.pdf]

Description of Additional Supplementary Files

File name: Supplementary data 1

Description: Microsatellite data given in fragment lengths with sample site and sampling year indicated. Missing data is represented with 0.
