# Supplementary material for: Muskrats as a bellwether of a drying delta
Source: Commun Biol. 2021 Jun 24;4:750. doi: 10.1038/s42003-021-02288-7 (PMC8225612; doi:10.1038/s42003-021-02288-7)
Supplement: Supplementary file 4 — Reporting Summary [file 42003_2021_2288_MOESM4_ESM.pdf]

nature research

Corresponding author(s): Ellen Ward

Last updated by author(s): May 21, 2021

Reporting Summary

Nature Research wishes to improve the reproducibility of the work that we publish. This form provides structure for consistency and transparency in reporting. For further information on Nature Research policies, see our Editorial Policies and the Editorial Policy Checklist.

Statistics

For all statistical analyses, confirm that the following items are present in the figure legend, table legend, main text, or Methods section.

n/a	Confirmed	
☐	☒	The exact sample size (*n*) for each experimental group/condition, given as a discrete number and unit of measurement
☐	☒	A statement on whether measurements were taken from distinct samples or whether the same sample was measured repeatedly
☐	☒	The statistical test(s) used AND whether they are one- or two-sided<br>*Only common tests should be described solely by name; describe more complex techniques in the Methods section.*
☒	☐	A description of all covariates tested
☐	☒	A description of any assumptions or corrections, such as tests of normality and adjustment for multiple comparisons
☐	☒	A full description of the statistical parameters including central tendency (e.g. means) or other basic estimates (e.g. regression coefficient) AND variation (e.g. standard deviation) or associated estimates of uncertainty (e.g. confidence intervals)
☐	☒	For null hypothesis testing, the test statistic (e.g. *F*, *t*, *r*) with confidence intervals, effect sizes, degrees of freedom and *P* value noted<br>*Give P values as exact values whenever suitable.*
☐	☒	For Bayesian analysis, information on the choice of priors and Markov chain Monte Carlo settings
☒	☐	For hierarchical and complex designs, identification of the appropriate level for tests and full reporting of outcomes
☒	☐	Estimates of effect sizes (e.g. Cohen's *d*, Pearson's *r*), indicating how they were calculated

Our web collection on statistics for biologists contains articles on many of the points above.

Software and code

Policy information about availability of computer code

Data collection	Agent modeling data were generated using the open source modeling framework HexSim Version 4.0.13.0. All microsatellite fragment traces were scored using Geneious v7.1.4.
Data analysis	Agent modeling results were analyzed using the source-sink mapping and dispersal flux mapping capabilities of the open source modeling framework HexSim Version 4.0.13.0 and in R/v3.4.3. For cytochrome b sequence data, we calculated the number of haplotypes, the number of polymorphic sites, and the nucleotide and haplotype diversity using DnaSP v6.11.01. For microstatellite data, we investigated the presence of null alleles using MICROCHECKER v 2.2.3 and estimated the frequency of null alleles in each sampling site using genepop v1.0.5 in R. We calculated the number of alleles and allelic richness using FSTAT V2.9.3.2. We calculated the observed and expected heterozygosity and deviations from Hardy-Weinberg equilibrium using Arlequin 3.5.2.2. We assessed pairwise genetic difference between sites calculating RST in Arlequin 3.5.2.2. Population structure was assessed using the Bayesian clustering approach implemented in the program STRUCTURE v2.3.4. The rate of change in the log probability of the data between successive K values (Delta K) was calculated and visualized using STRUCTURE HARVESTER Web v0.6.94 and structure results were visualized using CLUMPAK. We identified putative first-order relatives (parent-offspring or full siblings) by cross referencing output from three different programs – Colony v 2.0.6.5, ML-Relate, and Coancestry. Effective population size (Ne) was estimated using NeEstimator and MLNe.

For manuscripts utilizing custom algorithms or software that are central to the research but not yet described in published literature, software must be made available to editors and reviewers. We strongly encourage code deposition in a community repository (e.g. GitHub). See the Nature Research guidelines for submitting code & software for further information.

April 2020

Data

Policy information about availability of data

All manuscripts must include a data availability statement. This statement should provide the following information, where applicable:

- Accession codes, unique identifiers, or web links for publicly available datasets
- A list of figures that have associated raw data
- A description of any restrictions on data availability

The agent-based model and modeling results, including raw data associated with figures 1, 2 and 3, are available at https://doi.org/10.17605/OSF.IO/CHNR6. All cytochrome b sequence data have been submitted to the GenBank database under accession numbers MT215718 - MT215954. Figure 2 has associated raw data from the genetics analyses; that microsatellite dataset is included as Supplementary Data 1. Muskrat tissue samples are available from E.A. Hadly upon reasonable request.

Field-specific reporting

Please select the one below that is the best fit for your research. If you are not sure, read the appropriate sections before making your selection.

☐ Life sciences ☐ Behavioural & social sciences ☒ Ecological, evolutionary & environmental sciences

For a reference copy of the document with all sections, see nature.com/documents/nr-reporting-summary-flat.pdf

Ecological, evolutionary & environmental sciences study design

All studies must disclose on these points even when the disclosure is negative.

Study description	Muskrat tissue samples donated by local trappers were used to study the genetics of muskrat in the Canadian Peace-Athabasca Delta. A total of 200 muskrat samples from 6 sites were donated from the 2015 trapping season and 88 muskrats from 4 sites were donated in the 2016 trapping season. We sequenced 872 base pairs of mitochondrial cytochrome b and genotyped 9 autosomal microsatellite loci for these samples. We used this genetic data to assess population structure, relatedness among samples, and effective population size. Additionally, this study involved agent-based modeling of the muskrat population in the delta over the period 1972-2017. Model output was compared to the genetics results and used to assess changes in muskrat abundance over time as well as carry out source-sink and muskrat dispersal mapping for target years.
Research sample	The genetic samples consisted of tissue samples of muskrat (Ondatra zibethicus) from the Canadian Peace-Athabasca Delta donated by local trappers. Sex and age of the muskrat sampled are unknown. A total of 200 muskrat samples from 6 sites were donated from the 2015 trapping season and 88 muskrats from 4 sites were donated in the 2016 trapping season. We had no control over the number of samples donated nor the specific location within the delta where samples were collected.
Sampling strategy	This study was designed to capitalize upon muskrat trapping that was already taking place in the delta, thus, we had no control over the number of samples donated in each year. In the end, a total of 200 muskrat samples from 6 sites were donated from the 2015 trapping season and 88 muskrat samples from 4 sites were donated in the 2016 trapping season. As shown in the haplotype curve in Extended Data Figure 4, the 200 samples collected in 2015 were more than what was required to capture all cytochrome b haplotype diversity present, so only 150 of these samples were sequenced.
Data collection	Local trappers recorded sample location data for tissue samples. All samples were PCR amplified, fragment analyzed, and scored twice by Katherine Solari using Geneious v7.1.4.
Timing and spatial scale	Muskrat tissue samples for this study consisted of <2mm diameter pieces of tail tissue donated by trappers from muskrats trapped for fur and meat during the 2015 and 2016 trapping seasons (November to May). All muskrats were collected from the Peace-Athabasca Delta. As samples were donated by trappers, we had no control over when or where within the delta samples were collected.
Data exclusions	All samples were PCR amplified, fragment analyzed and scored twice to assure genotyping accuracy. All scoring was conducted in Geneious v7.1.4. Three samples from 2015 were removed due to missing data and/or discrepancies between the two genotyping runs. Sample site G was excluded from our calculations of RST as this was the only site with less than 10 samples.
Reproducibility	The reproducibly of microsatellite data was tested by amplifying, fragment analyzing and scoring every sample twice. Three samples from 2015 were removed due to missing data and/or discrepancies between the two genotyping runs. In our final dataset, 90.3% of alleles from 2015 were confirmed by two identical genotypes and 96.5% of alleles from 2016 were confirmed by two identical genotypes. The remainder were alleles that were successfully captured in only one fragment analysis and were missed in the other run due to allelic dropout.
Randomization	The genetics aspect of this study was an observational study of the natural distribution of genetic diversity of muskrat across the delta, thus, samples were grouped based on their geographic location.
Blinding	We had no hypothesis regarding the distribution of genetic diversity in muskrat across the delta going into this study, thus, blinding was not relevant.

Did the study involve field work? ☐ Yes ☒ No

Reporting for specific materials, systems and methods

We require information from authors about some types of materials, experimental systems and methods used in many studies. Here, indicate whether each material, system or method listed is relevant to your study. If you are not sure if a list item applies to your research, read the appropriate section before selecting a response.

Materials & experimental systems

n/a	Involved in the study
☒	☐ Antibodies
☒	☐ Eukaryotic cell lines
☒	☐ Palaeontology and archaeology
☒	☐ Animals and other organisms
☒	☐ Human research participants
☒	☐ Clinical data
☒	☐ Dual use research of concern

Methods

n/a	Involved in the study
☒	☐ ChIP-seq
☒	☐ Flow cytometry
☒	☐ MRI-based neuroimaging
